# Empathy as a Function of Clinical Exposure - Reading Emotion in the Eyes

**DOI:** 10.1371/journal.pone.0065159

**Published:** 2013-06-05

**Authors:** Cameron Handford, Jim Lemon, Michael C. Grimm, Ute Vollmer-Conna

**Affiliations:** 1 School of Psychiatry, University of NSW, Sydney, New South Wales, Australia; 2 St George Clinical School, University of NSW, Sydney, New South Wales, Australia; University of Bologna, Italy

## Abstract

**Background:**

Evidence based largely on self-report data suggests that factors associated with medical education erode the critical human quality of empathy. These reports have caused serious concern among medical educators and clinicians and have led to changes in medical curricula around the world. This study aims to provide a more objective index of possible changes in empathy across the spectrum of clinical exposure, by using a behavioural test of empathic accuracy in addition to self-report questionnaires. Moreover, non-medical groups were used to control for maturation effects.

**Methods:**

Three medical groups (N = 3×20) representing a spectrum of clinical exposure, and two non-medical groups (N = 2×20) matched for age, sex and educational achievements completed self-report measures of empathy, and tests of empathic accuracy and interoceptive sensitivity.

**Results:**

Between-group differences in reported empathy related to maturation rather than clinical training/exposure. Conversely, analyses of the “eyes” test results specifically identified clinical practice, but not medical education, as the key influence on performance. The data from the interoception task did not support a link between visceral feedback and empathic processes.

**Conclusions:**

Clinical practice, but not medical education, impacts on empathy development and seems instrumental in maintaining empathetic skills against the general trend of declining empathic accuracy with age.

## Introduction

The ability to understand and connect with the emotional state and mind frame of another is referred to as empathy and is thought to be a multidimensional construct encapsulating elements of both affective and cognitive processing [1], [2]. In no other setting is empathy more paramount than in the medical field in the context of patient care [3]. An accumulation of recent findings suggests that the current structure of medical education and the pervading model of health care delivery erode the innate predisposition to empathize [4]–[9]. The reasons offered by different authors for the apparent decline in empathy across medical training are still speculative and no agreement as to causal relationships has been reached. Several authors have suggested that the decline in empathy may be related to increasing levels of psychological distress or pressure to perform [4], [9]–[11]; others have postulated an escalation of cynicism and a ‘hardening of the heart’ reflect learned coping or survival mechanisms [4], [6]. However concerns over the methodological validity of these reports highlight the need for further research into empathy in the medical field [12]. Research investigating empathy levels in medical students and doctors needs to broaden its perspectives beyond the self-report questionnaire [13]. Such questionnaires do not provide optimal assessment of empathy as indicated by the lack of correlation with third person assessment of medical students’ empathy [14]. Methodologically critical is the absence of control groups in studies using self-report questionnaires to show a decline in empathy in medical students and doctors [4]–[9]. Thus, from the available evidence it cannot be concluded that the observed changes in self-reported empathy are due to medical training rather than general moral development [15].

A more objective method of assessing empathy is offered by emotion recognition tasks [16]. Facial expressions have a major role to play in emotion communication [17]. Evolutionary theorists propose that humans have evolved so that emotions can be rapidly communicated through facial expressions [18]. This is supported by evidence related to the universal nature of facial expressions whereby even the least technologically advanced societies recognize expressions that accompany the basic emotions, including happiness, sadness, anger, fear and disgust [19]. Furthermore, it is the eye region alone which is reported to convey most of the information regarding the emotional state of the individual [16], [20]. The “Reading the Mind in the Eyes” test utilizes an understanding of subtle mental states and how to recognize them in another [21]. This test has been classified as a measure of cognitive empathic accuracy [22]. Cognitive empathy is the conscious process of vicariously taking the perspective of another and utilizing learned information to infer the emotional state of another. Other studies have demonstrated that there are visceral and affective processes involved in emotion recognition [23], [24]. Interoception tasks, in which an awareness of the physiological state of the body is assessed [25], [26], provide a means to investigate the visceral component of empathy [27]. A specific link between the “eyes” test and the visceral components of empathy has not been investigated.

The present study had two objectives, the first was to re-examine the putative decline in empathy across the different stages of medical training and clinical exposure, using a behavioural measure of empathic accuracy in addition to the conventional self-report instruments. A second objective was to compare the results obtained from the medical groups to appropriately matched control groups. Additionally, an interoception task was utilized to investigate the putative contribution of affective visceral processes to empathic accuracy. Our results demonstrate significant complexity in the maturation of empathic processes in clinicians.

## Methods

### Participants and Study Design

Participants were recruited from the University of New South Wales (UNSW), affiliated teaching hospitals, and the general community via email, social networking sites and posters. There were five participant groups: three medical and two control groups. The UNSW medical course is six years in duration and is divided into three two-year phases. Twenty (11 females) MedPhase1 students and 20 (10 females) MedPhase3 students participated in the study. The final medical group consisted of 20 (9 females) registered medical practitioners across a spectrum of medical specialties affiliated with UNSW (mean duration of clinical practice 22 years). There were two control groups, each containing 20 participants. One consisted of non-medical students of similar age and sex distribution (10 females) as the participating medical students. For appropriate comparisons with the doctors involved in the study, we recruited an ‘older’ control group of similar age, sex ratio (9 females) and educational achievements (i.e., academics in other disciplines, and professionals). To restrict potential confounds on performance of the behavioural task, as well as on heart rate, which was monitored via electrocardiography (ECG) during the interoception task, exclusion criteria for the study were: pregnancy, primary sleep disorder, significant impairment of vision and/or hearing, endocrine, neurological, autoimmune or cardiovascular disease and any major psychiatric or substance abuse disorders. Medications including beta-blockers, benzodiazepines, corticosteroids and any other centrally active drugs were also exclusionary.

### Ethics Statement

The relevant Human Research Ethics Committee of the University of NSW approved this research (Approval No: HREA10020). The study was conducted in accordance with the principles expressed in the Declaration of Helsinki. All participants gave written informed consent before taking part.

### Procedure

Testing was carried out at a comfortable ambient temperature (23±3°C) under controlled laboratory conditions. Participants were asked to abstain from caffeine, alcohol and exercise for 12 hours prior to testing as these could affect heart rate and confound results on the interoception task. Upon arrival in the laboratory relevant medical and demographic information were recorded; and standardized questionnaires were completed to provide information regarding health behaviour, psychological state and traits, and self-reported empathy. Participants were then connected to physiological sensors (consisting of a 3 lead ECG) for heartbeat detection accuracy test. Following this, participants performed a modified computer version of the “Reading the Mind in the Eyes” test (see details below).

### Questionnaires

Participants answered questionnaires regarding personality and psychological state, and completed two standard self-report instruments specifically pertaining to empathy - the Interpersonal Reactivity Index (IRI) [28] and the Empathy Quotient [1]. Additional questionnaires used in the study were the Kessler 10 (K10) psychological distress scale [29], which provides a global measure of emotional state based on common symptoms of anxiety and depression; the Perceived Stress Questionnaire (PSQ) [30], a 30-item questionnaire which quantifies current levels of life stress; and the short form of the Eysenck Personality Questionnaire [31] to measure relevant aspects of personality, in particular neuroticism and extroversion.

### Emotion Recognition Task

Subjects performed a modified version of the “Revised Reading the Mind in the Eyes” test (referred to as the “eyes” test) [21]. The test consists of a series of photographs of the eye region of actors/actresses displaying different emotions. The images were displayed on a computer screen for three seconds and participants were asked to select the most appropriate of four possible descriptors for the emotion depicted in the eyes (e.g. *serious*, *ashamed, alarmed and bewildered*). No feedback was provided and the answers were recorded electronically for later analysis. To ensure that all participants were aware of the meaning of the words used in the “eyes” test a list of definitions was provided for all the descriptors used in the test prior to testing. We asked each participant to read through the list carefully. If any of the words were unfamiliar, participants were encouraged to study the definition and clear up any difficulties with the experimenter. Because our initial pilot testing suggested a ceiling effect in the “eyes” test performance of both medical and non-medical members of our highly intelligent target groups, we have modified this test by limiting the display time of the stimuli to three seconds and by adding a distractor task. Specifically, participants were required to press a ‘red button’ whenever they heard a distinctive target tone (a beeping signal explained as an alarm) mixed in a background hospital soundscape. The hospital theme was used to focus the subjects’ attention on the distractor task, i.e., identifying and responding to the beeping signal. The hospital sounds formed part of the plausibility of this distractor task. The sounds themselves were not unusual (i.e., telephones, beepers, alarms, and voices). The original version of the “Reading the Mind in the Eyes” test [21] includes 36 different images/items. To get a clear estimation of participants’ ability on the “eyes” test, each item of the test was presented twice in randomized order. Due to a technical error, the data relating to one of the images (i.e., Item 36: ashamed, nervous, *suspicious,* indecisive) was not consistently recorded and the analyses were therefore based on results obtained from 2×35 trials.

### Heart Beat Detection Task (Interoception)

A ML880 16 channel PowerLab using Labchart Pro7 software (ADInstruments, Bella Vista, Australia) was used to monitor HR without visual feedback to the subject, with the onset of each pulse waveform triggering a tone. This task was based on the Method of Constant Stimuli [32]. Twenty-eight trials were played, each involving delivery of a set of 10 tones, which were either ‘synchronous’ to the individual’s heartbeat or delayed to occur exactly at the midpoint of the R-R interval. Subjects attended to their own heartbeat and indicated at the end of each trial whether the feedback was synchronous or delayed. A heartbeat detection accuracy score was calculated by dividing the number of correct responses by the total number of trials.

### Statistical Analyses

Statistical analyses were performed using PASW Statistics for Windows version 18 (SPSS Inc., Chicago, IL, USA). The sample size was estimated to demonstrate a medium effect size on major outcome variables at 80% statistical power and α = 0.05. The dataset was complete. Normality of variables was ascertained by graphical methods (Q-Q plots). Between-group differences in empathy measures and other relevant variables were tested using contrast analysis within a one-way ANOVA model. The contrasts were used to estimate specific group effects, for example, a main effect for “medical participants” [all medical participants (Meds) versus all control subjects (Controls)], or a linear trend for clinical experience (a consistent change from MedPhase1 to MedPhase3 to Doctors). Mann-Whitney U tests were used to compare health behaviour data as these were not normally distributed. Correlation (Pearson) analyses served to assess bivariate associations, and Chi-square tests assessed independence of categorical data. To evaluate the relative contribution of key predictors and covariates to outcomes on the emotion recognition test multiple regression analysis was used.

## Results

### Participant Characteristics

There were no significant differences between the medical groups and the relevant control group in regard to age, sex, BMI, personality measures and current levels of perceived life stress or emotional distress (**Table 1**). The medical and control participants did not differ in terms of median caffeine intake [Controls: 2 (minimum-maximum: 0–5 cups/day); Meds: 1 (0–6); *P* = 0.27]; or the median number of cigarettes smoked per day [overall 6% smoked; Controls: 0 (0–10); Meds: 0 (0–5); *P* = 0.17]. Control participants consumed more alcohol [median number of standard drinks/week; Controls: 3 (0–25); Meds: 1 (0–20); *P* = 0.02] and engaged in more exercise [Controls: median hours/week 5.5 (0–23); Meds: 4 (0–14); *P* = 0.04] than their medical counterparts.

**Table 1:** Characteristics of the five participant groups including age, sex, BMI, personality aspects and current levels of life stress and distress.

	Medical Students		Controls		Doctors	Controls	
	MedPhase1	MedPhase3	Younger	*p-value* *		Older	*p-value*
**Age**	19.2 (1.2)	23.2 (1.2)	21.6 (1.4)	0.84	45.9 (9.5)	44.4 (13.7)	0.69
**Female:Male** **ratio**	11∶9	12∶8	10∶10	0.58	9∶11	9∶11	0.55
**BMI (kg/m^2^)**	21.6 (2.6)	22.2 (3.2)	22.7 (3.5)	0.47	25.2 (4.3)	26.0 (5.7)	0.65
**Extraversion**	8.0 (3.3)	6.2 (3.4)	7.3 (4.3)	0.85	7.5 (4.4)	6.9 (5.1)	0.72
**Neuroticism**	5.4 (2.9)	3.6 (2.5)	4.6 (3.4)	0.90	2.5 (2.8)	3.7 (2.9)	0.22
**Distress** (K10 score)	16.3 (5.4)	13.5 (4.0)	15.0 (3.2)	0.92	14.2 (5.6)	15.0 (5.1)	0.62
**Life stress** (PSQ score)	62.5 (13.6)	59.9 (13.7)	61.4 (14.8)	0.98	61.5 (18.3)	62.9 (16.3)	0.80

(Values are group means and standard deviations in parenthesis, n = 20 per group).

BMI = body mass index; PSQ = Perceived Stress Questionnaire; K10 = Kessler 10.

* A p-value was obtained by comparing the combined mean of the two medical student groups to that of the younger control group.

### Questionnaires

**Figure 1:**
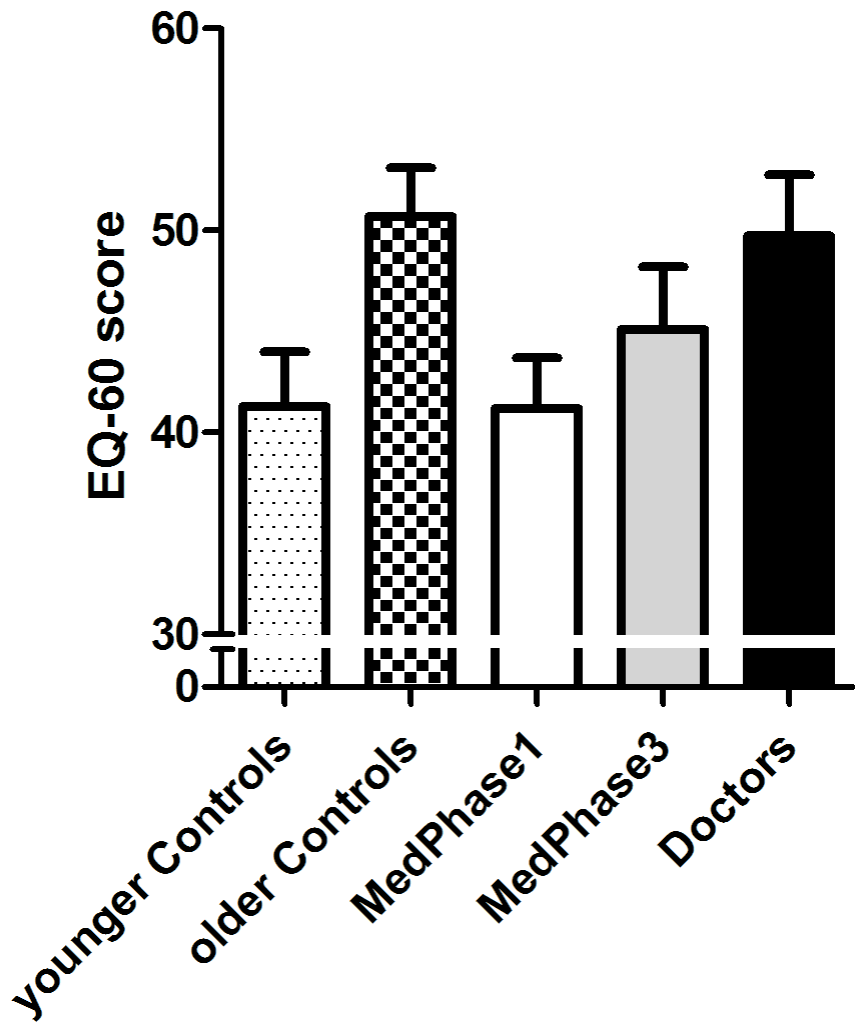
Mean scores for the Empathy Quotient questionnaire (EQ-60) for the five participant groups. Error bars represent the standard error of the mean.

**Figure 2:**
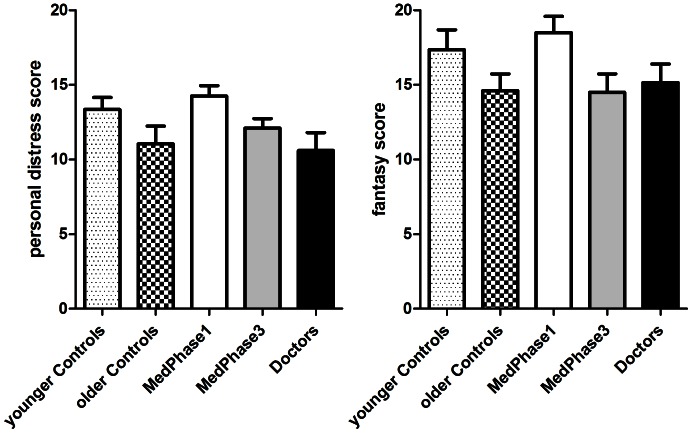
Mean scores for the *personal distress* (A) and *fantasy* (B) subscales of the Interpersonal Reactivity Index (IRI), for the five participant groups. Error bars represent the standard error of the mean.

Mean scores on the different empathy measures are reported for the five participant groups in **Table 2**. The questionnaire data revealed a similar pattern of results across several empathy measures - the EQ-60 and the IRI subscales *empathic concern* and *perspective taking*. Notably, the average scores showed a trend related to age with higher scores obtained by those of older age. In the scores obtained from the EQ-60 (see **Figure 1**) there was no difference between the medical groups overall (M = 45.3, SD = 13.2) and the control groups (M = 46.0, SD = 12.2) [F(1,95) = 0.07, *P* = 0.80]. However there was a significant linear trend across the medical groups with an increase in empathy scores related to medical training and practice [F(1,95) = 4.84, *P* = 0.03]. This was matched by a significant age related increase in the control groups [F(1,95) = 6.8, *P* = 0.01]. Although the scores on *empathic concern* and *perspective taking* showed very similar trends, these did not reach statistical significance. The scores on both *personal distress* and *fantasy* subscales of the IRI showed an inverse trend related to age (**Figures 2A&B**). Specific between-group comparisons for scores on the *personal distress* subscale (**Figure 2A**) showed no significant difference between the medical groups overall (M = 12.2, SD = 4.1) compared to those obtained by the control groups [M = 12.3 SD = 4.6; F(1,95) = 0.02, *P* = 0.90]. However, there was a significant linear trend for scores obtained across the medical groups [F(1,95) = 7.3, *P* = 0.007)] with a progressive decrease noted throughout the stages of medical training and practice. A similar decline in *personal distress* score between the younger and the older control participants approached significance [F(1,95) = 2.9, *P* = 0.08)]. The scores on the *fantasy* subscale (**Figure 2B**) similarly showed no significant difference between the scores obtained by the medical groups overall (M = 16.0, SD = 5.5) and the control group [M = 16.0, SD = 5.6; F(1,95) = 0.005, *P* = 0.95]. A linear trend in the results across the medical groups [F(1,95) = 4.0, *P* = 0.05] approached significance with a marked decrease in scores evident across medical training and practice. Again, a similar decline in scores between the younger and the older control participants approached significance [F(1,95) = 2.56, *P* = 0.10].

**Table 2:** Mean scores (and standard deviations in parenthesis, n = 20 per group) obtained from the different empathy measures for the five participant groups.

	Medical Students		Controls	Doctors	Controls
	MedPhase1	MedPhase3	Younger		Older
**EQ-60**	41.2 (11.2)	45.1 (13.9)	41.3 (12.0)	49.8 (13.5)	50.7 (10.8)
**IRI**					
*empathic concern*	20.5 (3.9)	20.8 (3.5)	19.8 (4.3)	22.0 (3.9)	21.8 (3.4)
*perspective taking*	17.9 (6.0)	18.8 (3.3)	17.0 (4.5)	20.0 (4.2)	19.1 (4.3)
*personal distress*	14.3 (3.1)	12.1 (2.9)	13.4 (3.6)	10.6 (5.3)	11.1 (5.3)
*fantasy*	18.5 (4.8)	14.5 (5.5)	17.4 (5.9)	15.1 (5.6)	14.6 (5.1)

EQ = Empathy Quotient; IRI = Interpersonal Reactivity Index.

As suggested by the patterns of results there was a series of significant correlations between scores of these questionnaires and age. The EQ-60 showed a positive correlation with age [r(98) = 0.27, *P* = 0.006]. The *personal distress* [r(98) = −0.33, *P* = 0.001] and *fantasy* [r(98) = −0.27, *P* = 0.006] subscales correlated negatively with age. The similar patterns seen across EQ-60, and the subscales *empathic concern* and *perspective* with a distinctly different pattern reflected in the scores for the *personal distress* and *fantasy* subscales were supported by differential correlations with personality measures. Extroversion significantly correlated with both the EQ-60 [r(98) = 0.32, *P* = 0.001] and *empathic concern* [r(98) = 0.26, *P* = 0.01]; whereas neuroticism significantly correlated with *personal distress* [r(98) = 0.41, *P*<0.001] and *fantasy* [r(98) = 0.31, *P* = 0.002].

### The “Eyes” Test

Reliability analyses of our version of the “eyes” test indicated moderate reliability, which is comparable to other versions of the test [33]. Specifically, tetrachoric correlation (for binary responses) was 0.55 with a tau coefficient of >0.6. A Pearson correlation of the sequence scores (number correct) for the first and second sequences yielded r(33) = 0.66. Overall performance on our variation of the “eyes” test (mean number correct items = 25.6) is almost identical to the normal population scores (26.2) on the standard version of this test [21]. Moreover repeated presentation of the test items did not lead to learning or practice effects. Repeated measures ANOVA revealed that across both presentations of the test stimuli, participants performed equivalently demonstrating no learning or practice effect [F(1,95) = 0.71, *P* = 0.4]. There was additionally no evidence of a differential practice effect as the interaction between groups and the repeated presentation of the test stimuli showed no significant interaction effect [F(4,95) = 0.39, *P* = 0.81].

**Figure 3:**
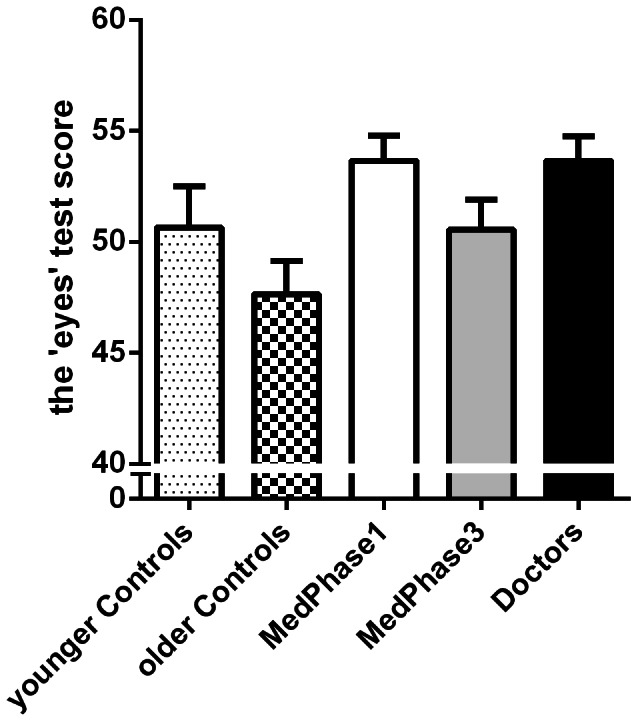
Mean scores for the “eyes” test for the five participant groups. Error bars represent the standard error of the mean.

Inspection of the results obtained for the “eyes” test (**Figure 3**) revealed a notably different pattern of results than those obtained from the self-report questionnaires. While there appears to be a trend related to age across the scores from the control groups, the doctors do not exhibit the same decline as older controls. Overall the medical group (M = 52.7, SD = 5.6) performed significantly better than the control group [M = 49.1, SD = 7.6; F(1,95) = 7.3, *P* = 0.009]. The noticeable decline in the scores between the younger control group (M = 50.7, SD = 8.3) and the older control group (M = 47.7, SD = 6.7) did not reach significance [F(1,95) = 2.3, *P* = 0.14]. There was no significant linear trend among the medical group [MedPhase1: M = 53.6, SD = 5.0; MedPhase3: M = 50.5, SD = 6.1; Doctors: M = 53.6, SD = 5.0]. However, a quadratic relationship did approach significance [F(1,95) = 3.8, *P* = 0.058] suggesting that performance on this test declined with medical training and then improved again during clinical practice.

Similar to the self-report data, a significant correlation with age was evident [r(98) = −0.27, *P* = 0.006], with increasing age relating to a decrease in performance. Performance in the “eyes” test correlated with scores on the EQ-60 (r(98) = 0.2, *P* = 0.049) and *fantasy* subscale [r(98) = 0.21, *P = *0.04]. In regards to personality measures the “eyes” test correlated significantly with extroversion [r(98) = 0.24, *P* = 0.02].

### Heartbeat Perception Accuracy (Interoception)

There were not significant differences between the groups on this measure [younger control group: M = 16.6, SD = 4.6; older control group: M = 14.9, SD = 3.6; MedPhase1: M = 15.5, SD = 3.2; MedPhase3: M = 14.6, SD = 4.9; and Doctors: M = 16.2, SD = 4.2; F(4,95) = 0.82, *P* = 0.52]. There were also no significant correlations between scores on this task and any of the relevant variables including the expected association between performance on the “eyes” test and heartbeat detection accuracy [r(98) = −0.06, *P* = 0.57].

### Predictors of Emotion Recognition Accuracy

Multiple regression modelling was employed to assess the relative importance of potential predictors of the performance in the “eyes” test, which were identified from earlier analyses and the literature. For inclusion in the model group membership reflecting clinical training/exposure was ‘dummy’ coded such that, for example, being a doctor or not constituted one dichotomous variable with 0 = no and 1 = yes. In addition to these variables, the model included age, sex, EQ-60 and fantasy sub-scale scores, personality variables (neuroticism and extroversion), current levels of life stress and emotional state. The model predicted 32% of the variance and was highly significant (R^2^ = 0.32, *P*<0.001). Being a doctor was the most important independent predictor of the ability to recognize emotion in the eyes (β = 0.45, *P*<0.001); specifically being a doctor was linked to an increase of almost ½ of a SD in the “eyes” test score. Young age was identified as the second most significant predictor (β = −0.38, *P* = 0.007) thus an increase in 1 SD in age led to a decline in the performance in the “eyes” test of 0.38 SD units. The score on the empathy questionnaire EQ-60 was also a significant independent contributor to emotion recognition (β = 0.22, *P* = 0.04).

## Discussion

This is the first study to assess empathy in medical students, clinicians and matched control participants that has employed a behavioural measure of empathic accuracy in addition to conventional self-report instruments. The results provided a number of novel insights into the dynamics of empathy. Firstly, neither of the methods used to investigate empathy supports the argument that medical education influences levels of empathy. Secondly, scores obtained from the self-report instruments are influenced by general developmental factors relating to age. Finally, the findings from this study document that experienced clinicians perform significantly better than age-matched controls of comparable professional standing; and further that clinical experience is the single most significant predictor of empathic accuracy. This offers an important and positive message that it is the human experience derived from actual doctor-patient interaction rather than medical education that counteracts an age-related decline in empathic accuracy.

### Strength and Limitations

The results obtained from the questionnaires in this study differ from previous findings in the literature which state that self-reported empathy declines throughout medical training [5], [7]–[9]. In contrast to previous findings, there was in fact no significant change in self-reported empathy that could be attributed to medical training and practice. An increase across the medical groups was evident on some measures (i.e., the EQ-60, and the *empathic concern* and *perspective taking* subscales of the IRI) but this matched an age-related increase seen in the control participants. That age was a critical factor here was further supported by the highly significant correlations with age and scores on these measures. Overall, these results strongly support an argument that changes in self-reported empathy are related to a cognitive/emotional maturation rather than the effects of clinical training.

Based on these observations it seems that as individuals mature, there is a shift in emotional responding from a self-focused response to a more externally- or other-focused emotional reactivity. This is supported by reports in the literature documenting that throughout emotional development from childhood to adulthood there is the continuous shift towards pro-social behaviour, where the wellbeing of others becomes more of a priority [15]. Specifically in regards to the medical profession, a study investigating psychological distress in older doctors found that they experienced less distress as they progressed through their careers [10]. Qualitative data from that study suggested the decrease in psychological distress was due to the development of protective mechanisms established throughout their career. While the current findings support this conclusion it should be noted that a similar effect was evident in non-medical participants. This underscores the importance of including appropriate non-medical control participants in study designs to permit correct interpretation of data relating to empathy among medical groups.

Medical groups as a whole performed better than the control groups in the emotion recognition task. However when comparing the medical groups to their respective control groups a more complex picture arose. The medical students performed no better than the younger control participants; however the doctors performed significantly better than the older control group. Regression modeling provided further insights into the determinants of emotion recognition accuracy. Being a doctor (with extensive clinical experience) and younger age emerged as powerful independent predictors. This suggests that the attributes of being a doctor prevent the age-related decline evident in the control groups. A tentative explanation could be that clinical practice requires doctors to decipher the emotional state of their patients in order to provide adequate medical care and therefore clinicians, by continuously exercising this skill, maintain this ability better than their non-medical peers.

Theories surrounding the ability to recognize emotion propose that societal pressures influence the accuracy with which people interpret facial expressions, for example, it is shown that individuals in subordinate positions perform better as, in order to satisfy their superiors, it is important to be able to accurately understand their emotional state [17]. The age group represented by the medical students and the younger control participants may coincided with increased social pressures to be accepted by one’s peers and thus accentuate the ability to read emotions accurately, leading to better performance on the “eyes” test. These societal pressures are generally less prevalent in the older population and, hence, their ability declines; however a doctor’s role requires them to maintain this ability. The objective behavioural data obtained via the “eyes” test present a dual message for the medical profession. A positive interpretation would be that clinical exposure and the years of experience associated with being a doctor maintains and develops the ability to read emotions accurately. Medical education itself and the pedagogic measures put in place to improve empathy appear to not have had a significant impact on increasing the cognitive or emotional aspects of empathy to date. In fact, the current data point towards a decline between early stages (Phase 1) and the later stages (Phase 3) of medical school. This is consistent with reports in the literature stating that the later stages of medical education and early clinical practice are critical periods, where peaks in cynicism, disillusionment and personal distress result in declines in empathy [10], [11]. The literature also reports that older, experienced doctors recover their empathic behaviour, which may be related to a concurrent decline in their personal distress [10].

A limitation of the current study in terms of generalization of findings is that all students were sampled from the same university and the medical students were all educated within the same (scenario based) curriculum. Moreover, while the “eyes” test offers an advance over previous studies in that it provides objective behavioural data of relevance to empathy, *in vivo* interaction with patients, although much harder to control, would permit a more realistic estimate.

Although our study included 100 participants, numbers within each subgroup were relatively small (N = 20). This may have limited statistical power, so that more subtle effects could not be detected. For example, group means did not differ with regard to psychological distress, current life stress, and personality factors (extroversion and neuroticism); however analysis of individual differences showed clear correlations between scores on the extroversion and neuroticism subscales and different aspects of empathy. While personality factors did not emerge as independent predictors in our multiple regression analysis, data from a larger sample may have revealed a role for personality in empathy and emotion recognition. Similarly, differences in psychological distress have been postulated by several authors [4], [10], [11] as instrumental in reducing empathy scores in medical students. Although this is a plausible assumption, we found no evidence to support such a link.

### Unanswered Questions and Future Research

No significant findings relating to interoceptive sensitivity as measured by the heartbeat detection task were obtained in this study. One explanation of this outcome is that the particular task employed is notoriously difficult with few participants achieving a score substantively above chance [34]. In future studies it would be of interest to use direct methods of monitoring the activation in interceptive brain regions (e.g., the insula and orbitofrontal cortex) via heartbeat-evoked brain potentials, or brain imaging via fMRI during emotion recognition tasks to more optimally examine the importance of interoceptive processing in empathic phenomena [26].

In addition, in the interest of the medical community, future studies should investigate the value of emotion recognition in relation to other constructs such as compassion and sympathy, which reflect more the behavioural outcomes of experiencing empathy. It is likely that these constructs rather than empathy itself relate more directly to patients’ satisfaction with their doctors.

Future studies with larger samples additionally need to re-examine the putative link between psychological distress and empathy, as well as a possible role for personality aspects in empathy and emotion recognition in medical groups and appropriate control subjects.

As it stands, the study enables fresh insights into empathy as a general construct and more specifically into empathy in the setting of the medical profession. Using the conventional self-report method in empathy research and a novel measure of empathic accuracy, this study debunks the belief that empathy declines as a function of clinical exposure. Rather it suggests that the clinical exposure linked to working as a doctor helps to foster empathy. The current results document that the changes previously reported for scores in empathy questionnaires are likely to be related to maturation rather than clinical training.
